# Supplementary material for: Systems biology approach reveals a link between mTORC1 and G2/M DNA damage checkpoint recovery
Source: Nat Commun. 2018 Sep 28;9:3982. doi: 10.1038/s41467-018-05639-x (PMC6162282; doi:10.1038/s41467-018-05639-x)

Systems biology approach reveals a link between mTORC1 and G2/M DNA damage checkpoint recovery

Hsieh et al.

Supplementary Information-Table

Supplementary Table 1:The top network functions and their molecules identified from IPA

Network functions	Molecules	Score	#Molecules
Cell death and survival, cancer, organismal injury and abnormalities	AKT1,AR,BAD,BCL2L1,BCL2L11,BECN1, CAV1,CDKN1A,CDKN1B,CHEK1,EGFR, ERBB2,ERBB3,FOXM1,FOXO3,MAP2K1, MET,MSH6,MTOR,PARP1,PCNA,PIK3CA, PIK3R1,RAF1,RB1,RICTOR,RPS6, RPS6KA1,SRC,STMN1,YAP1,YBX1, YWHAB,YWHAE,YWHAZ	79	35
Cell death and survival, cellular development, cellular growth and proliferation	BAK1,BID,BRCA2,CASP7,CCNB1,CCND1, CDH1,CTNNB1,DVL3,EIF4EBP1,FASN, GAPDH,LCK,MAPK1,MAPK8,MAPK14, NFKB1,PDCD4,PECAM1,PRKCD,RPTOR, SETD2,SMAD4,SNAI2,SRSF1	47	25

Supplementary Table 2: Oligonucleotides used for qRT-PCR and ChIP-qPCR

Oligonucleotides for qRT-PCR

Gene name		Primer Sequence (5’-3’)	Product
PLK1	F	GGCAACCTTTTCCTGAATGA	103 bp
	R	TCCCACACAGGGTCTTCTTC	
CCNB1	F	TTGGGGACATTGGTAACAAAGTC	226 bp
	R	ATAGGCTCAGGCGAAAGTTTTT	
ACTB	F	GAGCACAGAGCCTCGCCTTT	113 bp
	R	TCATCATCCATGGTGAGCTG	

Oligonucleotides for ChIP-qPCR

Gene name		Primer Sequence (5’-3’)	Promoter region
PLK1	F	GTAACGTTCCCAGCGCCG	-60 ~ +63 bp
	R	CAGCTTCCCTGCAGTCACTG	
CCNB1	F	CCAATAAGGAGGGAGCAGTG	+86 ~ +187 bp
	R	GGACCTACACCCAGCAGAAA	

F: forward; R: reverse

Supplementary Information-Figure

Supplementary Figure 1

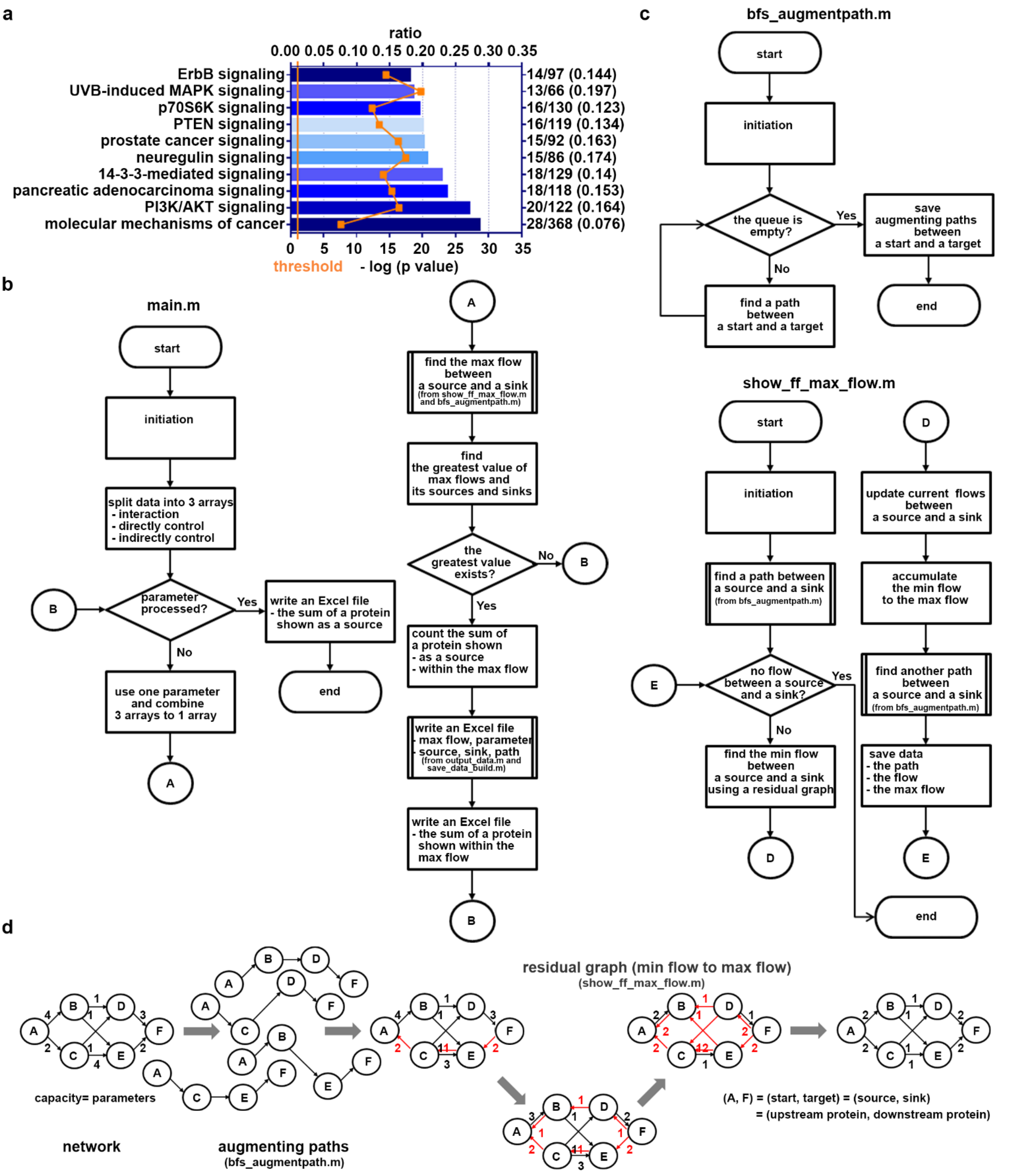

Supplementary Figure 1: The mathematics-based method is modified and applied for RPPA data analysis

a The top ten significant canonical pathways enriched with molecules from our screened RPPA dataset were calculated in IPA. The ratio indicated how many molecules in our RPPA dataset were associated with the specific pathway. **b**, **c** The flow charts demonstrate how we found potential targets and pathways in MATLAB. Briefly, we used the main program “main.m” to control subroutines (all other “.m” files). **d** The graph exemplifies how the Ford-Fulkerson algorithm works and how we defined the terms we used in our programs.

Supplementary Figure 2

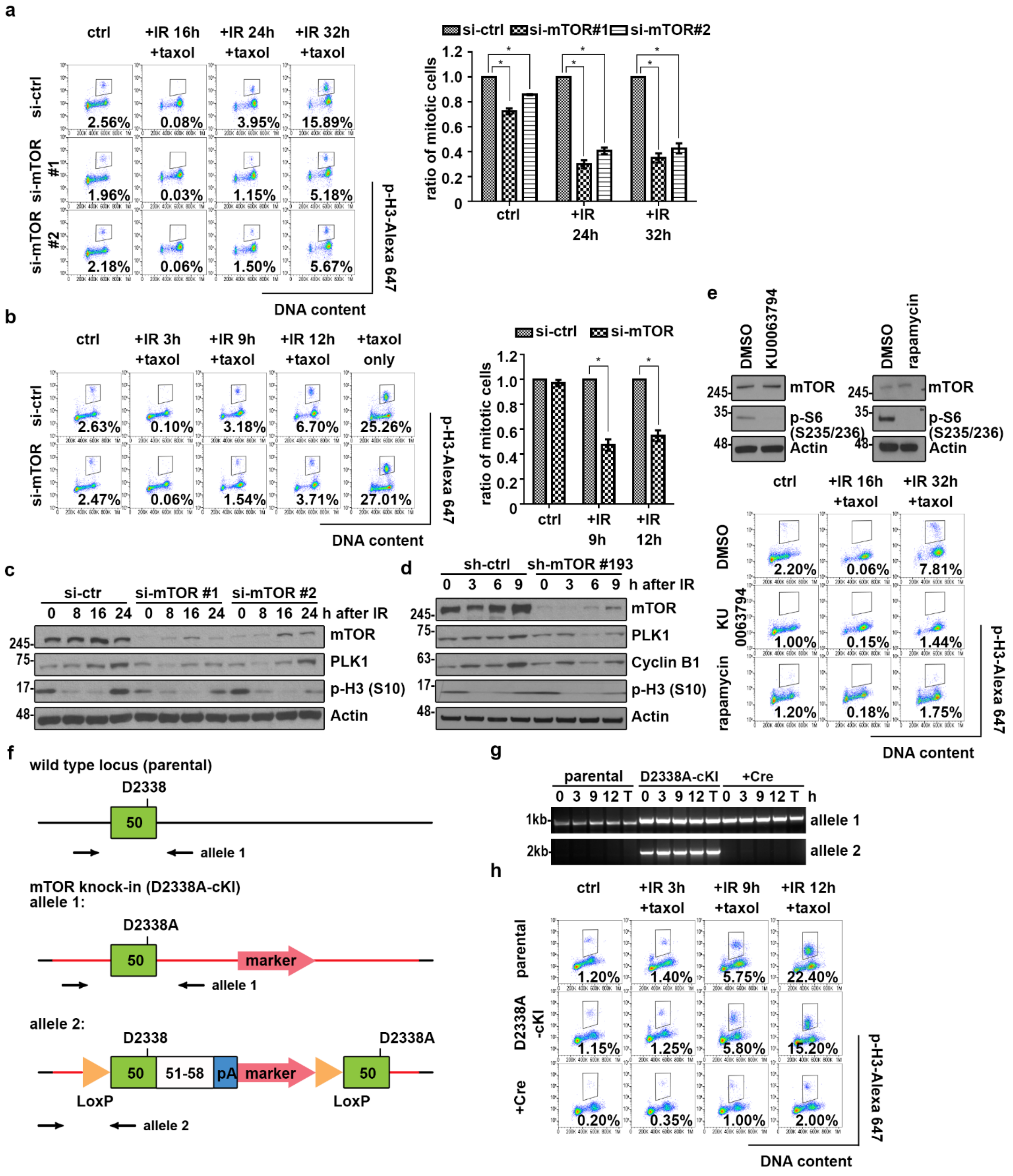

Supplementary Figure 2: The function of mTOR kinase in G2/M checkpoint recovery is not cell-type specific

a, **c** mTOR was depleted by two individual siRNA oligos (si-mTOR #1 and #2) in U2OS cells, and the experimental design was the same as Fig. 2c, e, and f. **b**, **d** We depleted mTOR by either shRNA or siRNA in HCT116 cells and treated cells with IR (7 Gy) plus 2 μM paclitaxel for Western blotting and mitotic entry analysis. **e** Efficiency of mTOR inhibition by rapamycin or KU0063794 was detected by Western blotting. The mitotic cell plots are representative plots of the results shown in Fig. 2g. The numbers indicate the percentages of p-H3–positive stained cells detected by flow cytometry. **f** The diagrams illustrate genomic structures of mTOR conditional knock-in cell line (D2338A-cKI) and primers (black arrows) identifying allele 1 and Cre-excised allele 2. **g** We followed the manufacturer's instructions to generate cells with mTOR-kinase–dead mutation (D2338A) using the Cre-Lox recombination system. PCR products on the agarose gel indicated efficiency of Cre recombinase. **h** The mitotic cell plots are representative plots of the results of the experiments described in Fig. 2h, i. si-ctrl and si-mTOR: cells transfected with non-target control siRNA and mTOR siRNA, respectively; ctrl: control; error bars: mean ± SEM; n = 3 independent experiments; *$p < 0.05$; two-tailed unpaired Student *t* tests

Supplementary Figure 3

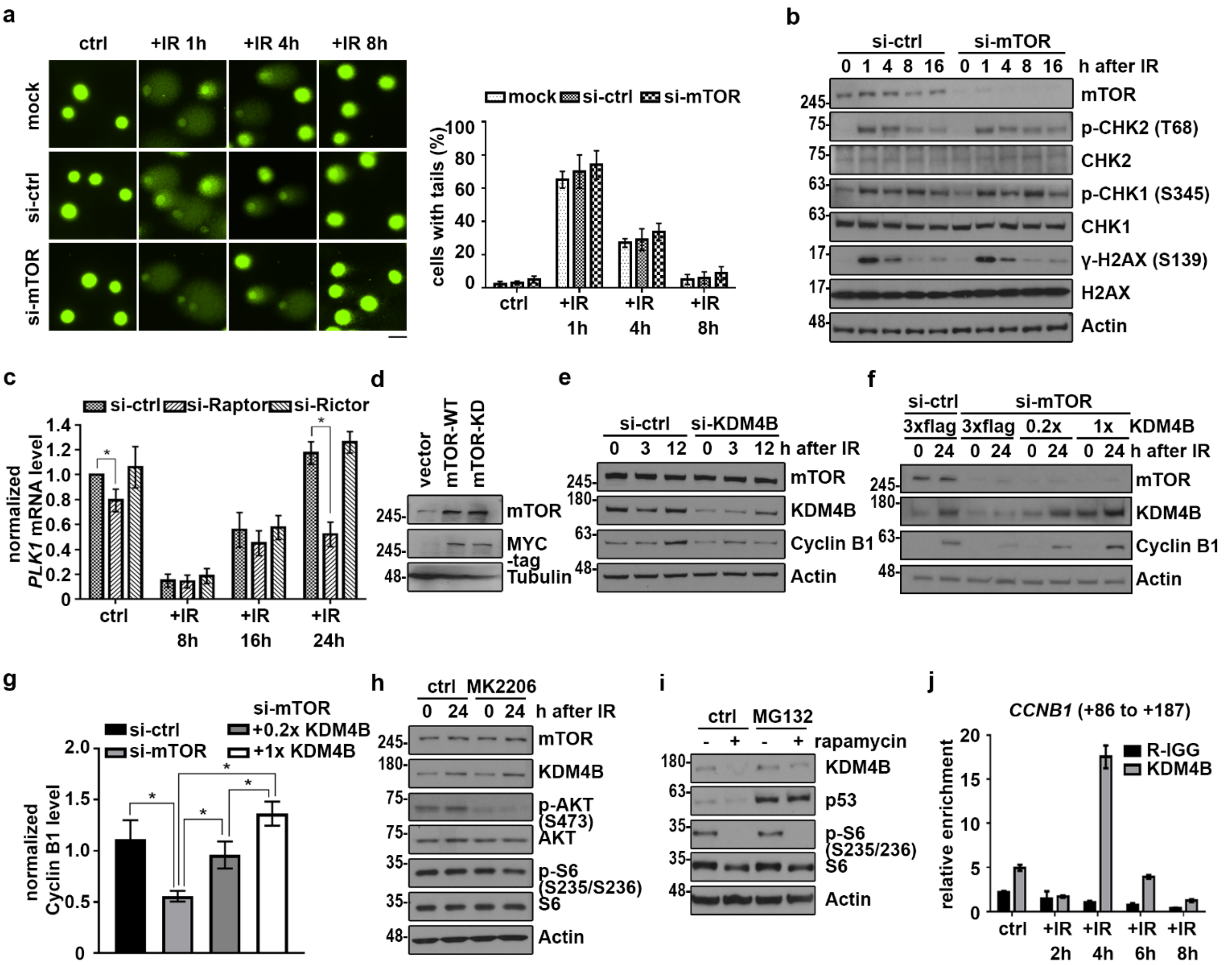

Supplementary Figure 3: mTOR does not affect IR-induced checkpoint activation and DNA damage repair but does regulate checkpoint recovery

a U2OS cells were transfected with or without siRNA and cells were collected at different time points after IR for the comet assay. Representative images showed the changes of comet tails after IR treatment. The scale bar indicates the distance is 10 µm. We counted 50 cells in each group, and the percentages of damaged cells in each group were analyzed. **b** U2OS cells transfected with siRNAs were collected after IR (7 Gy) for the kinetics of DNA damage response. **c** *PLK1* mRNA levels in U2OS cells transfected with siRNAs after IR (7 Gy) and 2 µM paclitaxel treatment were measured by quantitative reverse transcription–PCR as described in Fig. 3a, b. **d** Western blotting shows expression of the control vector, wild-type mTOR (mTOR-WT), and kinase-dead mTOR (mTOR-KD) constructs used in Fig. 3d. **e** We treated KDM4B-depleted HCT116 cells with IR (7 Gy) plus 2 µM paclitaxel for Western blotting. **f**, **g** Different amounts of KDM4B (0.2x or 1x) were overexpressed in mTOR-depleted U2OS cells and cells were then treated with IR plus paclitaxel for Western blotting. The graph in g is the quantitative results of cyclin B1 expression. **h** U2OS cells were pretreated with the AKT inhibitor MK2206 followed by IR and paclitaxel for Western blotting. **i** Rapamycin-pretreated U2OS cells were treated with the proteasome inhibitor MG132 to detect protein expression. **j** Quantitative ChIP at the *CCNB1* transcription regulation region was performed at different time points as described in Fig. 3i-k. Mock: cells incubated with only the transfection reagent; si-ctrl, si-Raptor, si-Rictor, and si-KDM4B: cells transfected with non-target control siRNA, Raptor, Rictor, and KDM4B siRNA, respectively; R-IGG: normal rabbit IgG; error bars: mean ± SD; n = 3 independent experiments; $*p < 0.05$; two-tailed unpaired Student *t* tests

Supplementary Figure 4

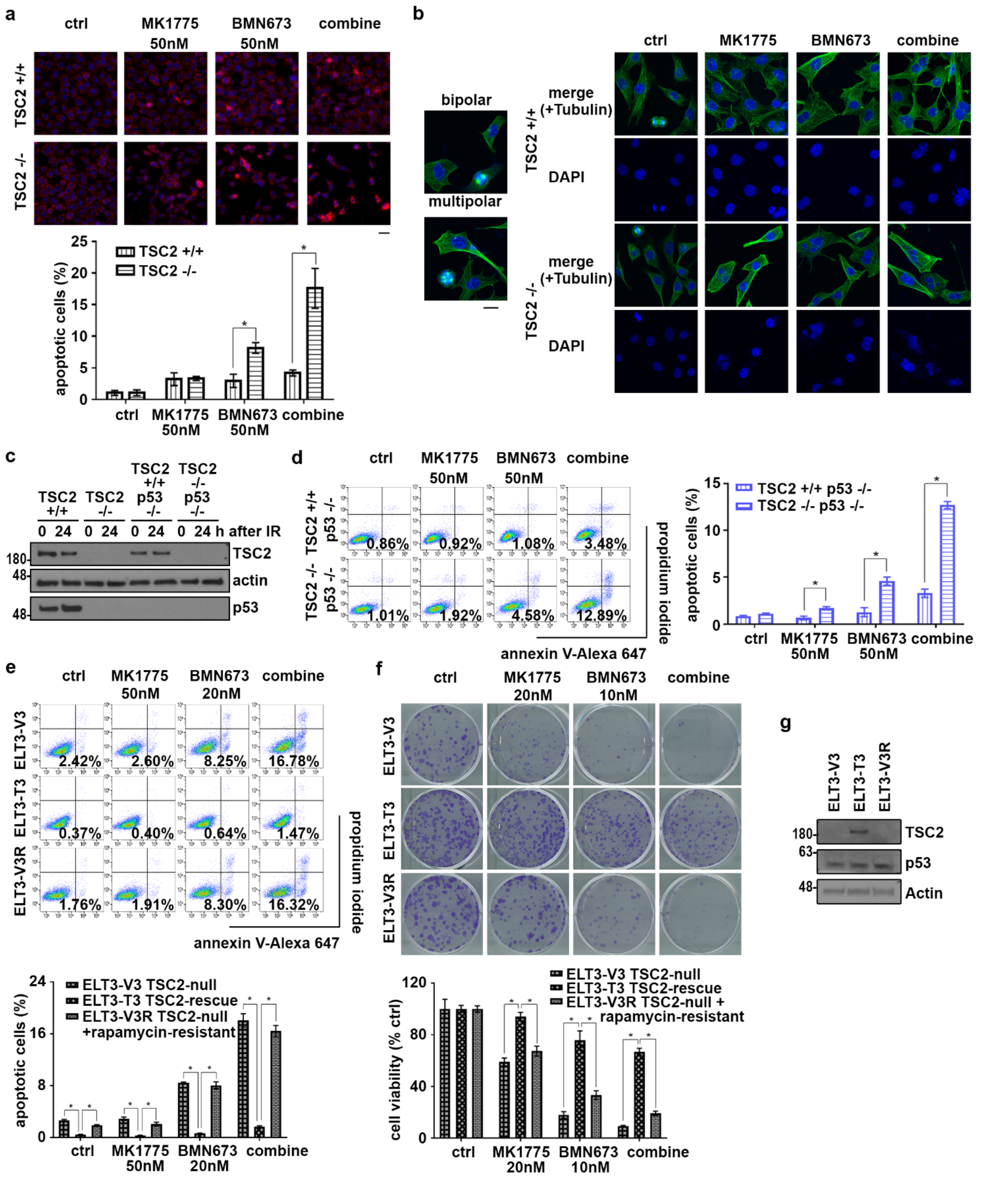

Supplementary Figure 4: TSC2-deficient cells are sensitive to the combination of MK1775 (the WEE1 inhibitor) with BMN673 (the PARP inhibitor)

a MEFs were treated as described in Fig. 4f and stained with cytochrome c. The percentages of apoptotic cells were calculated based on the positive staining of cytochrome c and nucleus morphology. The scale bar in the representative images is 20 µm. **b** The representative images for Fig. 4h are shown here. The scale bar indicates the distance is 10 µm. **c** Cells were exposed to IR (7Gy) for TSC2 and p53 protein expression. **d** MEFs were incubated with 50 nM MK1775 or/and 50 nM BMN673 for 60 hours for apoptosis assay. Quantitative results of apoptotic cells were shown in the bar graph. **e** ELT3 cells were treated with 50 nM MK1775 or/and 20 nM BMN673 for 72 hours and then subjected to apoptosis assay. **f** ELT3 cells were incubated with 20 nM MK1775 or/and 10 nM BMN673 for ten days for colony formation assay. Cell viability was the ratio of treated to untreated colonies in each line. **g** ELT3 cells were harvested directly without treatment for Western blotting. TSC2 +/+: $Tsc2^{+/+}$, Tsc2 wild-type; TSC2 -/-: $Tsc2^{-/-}$, Tsc2 null; p53 -/-: p53 deficient; ELT3-V3: Tsc2-null Eker rat uterine leiomyoma cells with the control vector; ELT3-T3: Tsc2-null ELT3 cells reexpressing *Tsc2*; ETL3-V3R: rapamycin-resistant ELT3-V3 cells; ctrl: control; error bars: mean ± SD; n = 3 independent experiments; *$p < 0.05$; two-tailed unpaired Student *t* tests

Supplementary Figure 5

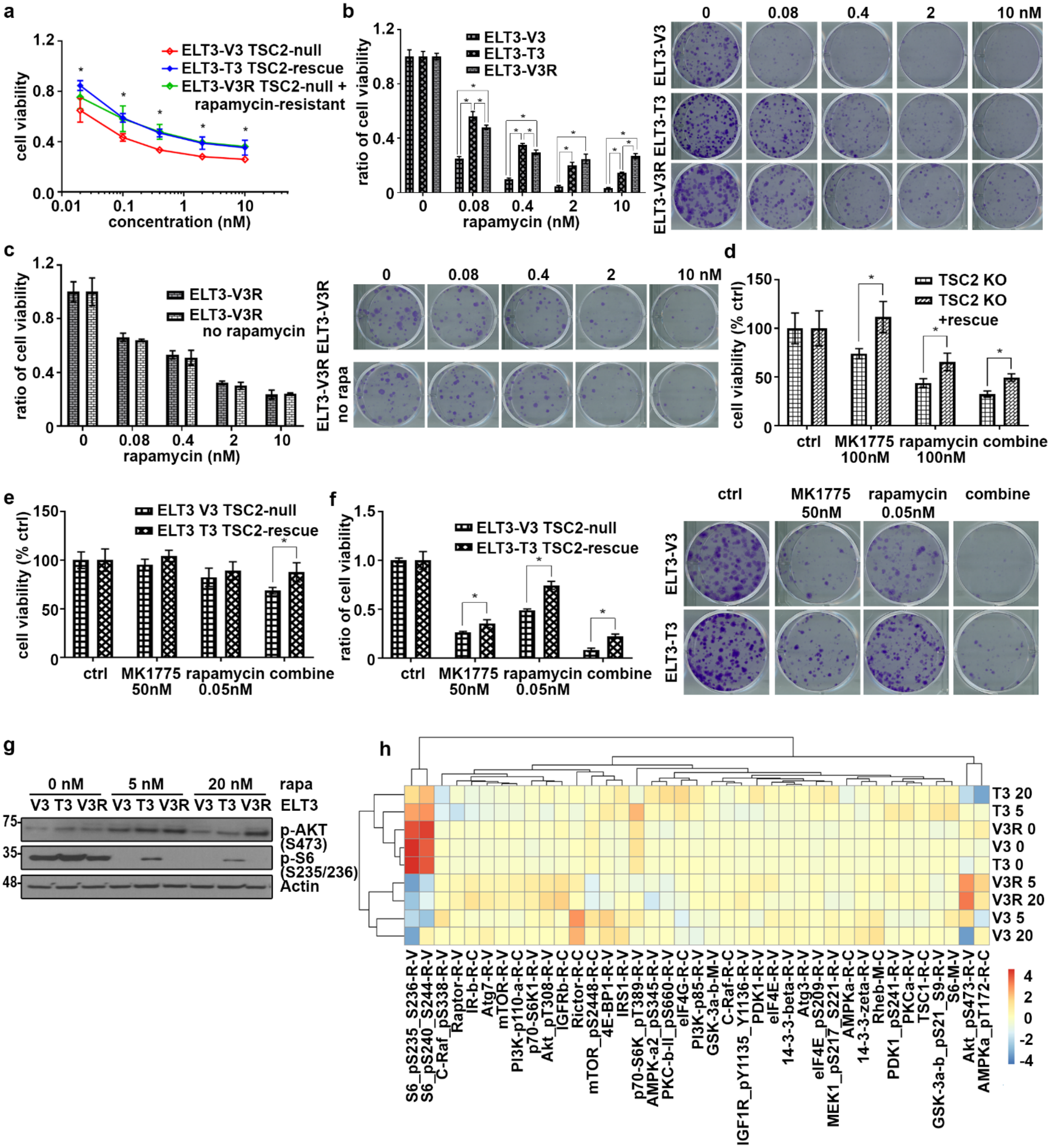

Supplementary Figure 5: Rapamycin-resistant TSC2-deficient cells remain sensitive to WEE1 inhibition

a, **b** Rapamycin sensitivity in ELT3 cells used in Fig. 4i was measured by MTT assay and the colony formation assay. **c** Rapamycin was withdrawn from cell culture medium in the "ELT3-V3R no rapa" group for two weeks before the colony formation assay. **d**, **e** MEFs and ELT3 cells were stained with trypan blue and counted after two-day drug treatment. The graphs represent the ratio of cell numbers to the control group in each cell line. **f** Drug sensitivity was measured by the colony formation assay. Cell culture media with indicated drugs were changed every three days. **g**, **h** ELT3 cells treated with different concentrations of rapamycin for 16 hours were collected for Western blotting and RPPA data analysis. Proteins related to the mTOR signaling pathway were analyzed. ELT3-V3: Tsc2-null Eker rat uterine leiomyoma cells with the control vector; ELT3-T3: Tsc2-null ELT3 cells reexpressing *Tsc2*; ETL3-V3R: rapamycin-resistant ELT3-V3 cells; TSC2 KO: $Tsc2^{-/-}$, Tsc2-null MEFs; TSC2 KO + rescue: TSC2 KO MEFs reexpressing *Tsc2*; ctrl: control; rapa: rapamycin; error bars: mean ± SD; n = 3 independent experiments; *$p < 0.05$; two-tailed unpaired Student *t* tests

Supplementary Figure 6-18: Uncropped Western blots

Supplementary Figure 6

Figure 2f

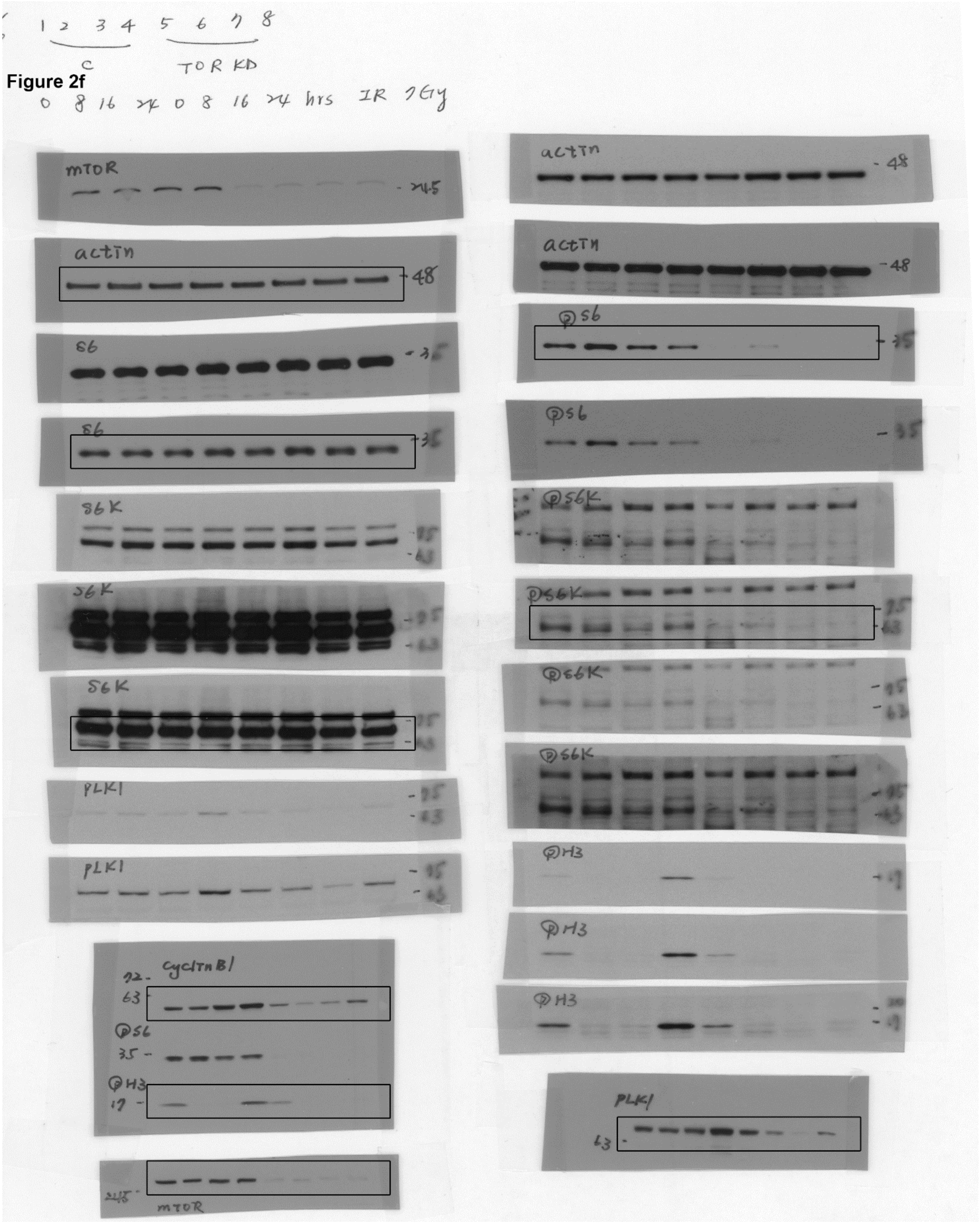

Supplementary Figure 7

Figure 2k

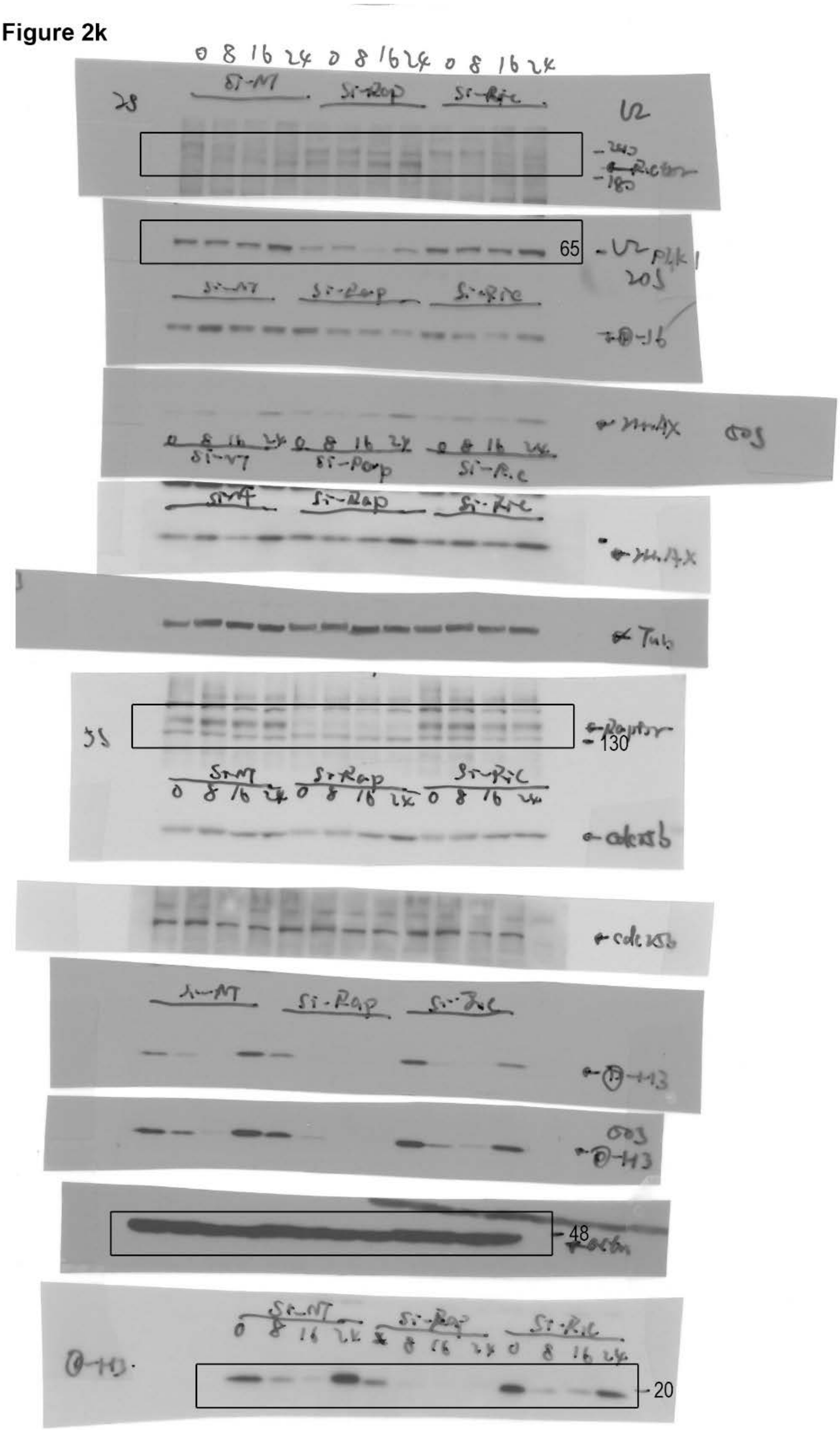

Supplementary Figure 8

Figure 2i

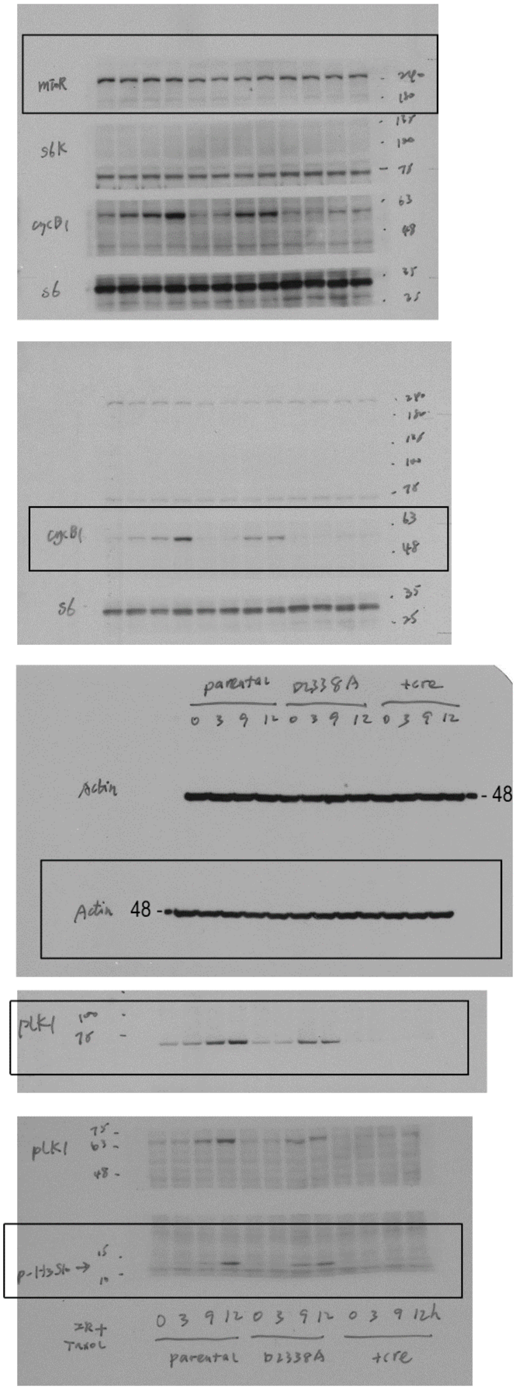

Figure 2b

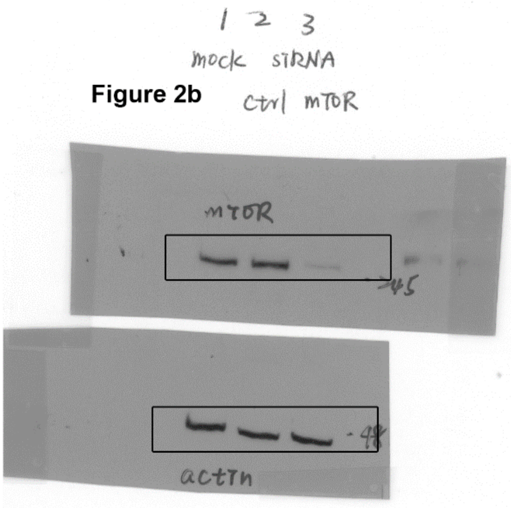

Supplementary Figure 9

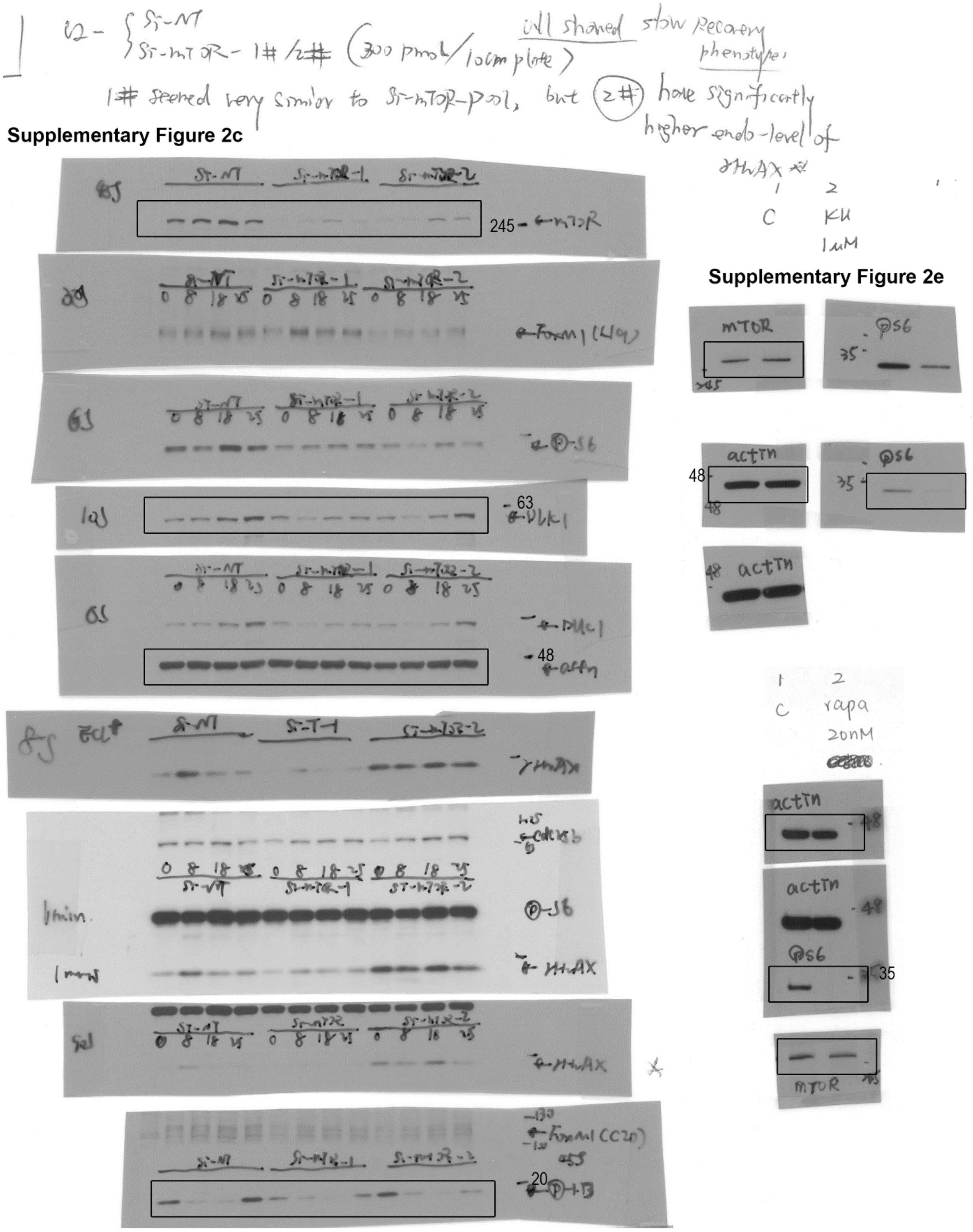

Supplementary Figure 10

Supplementary Figure 2d

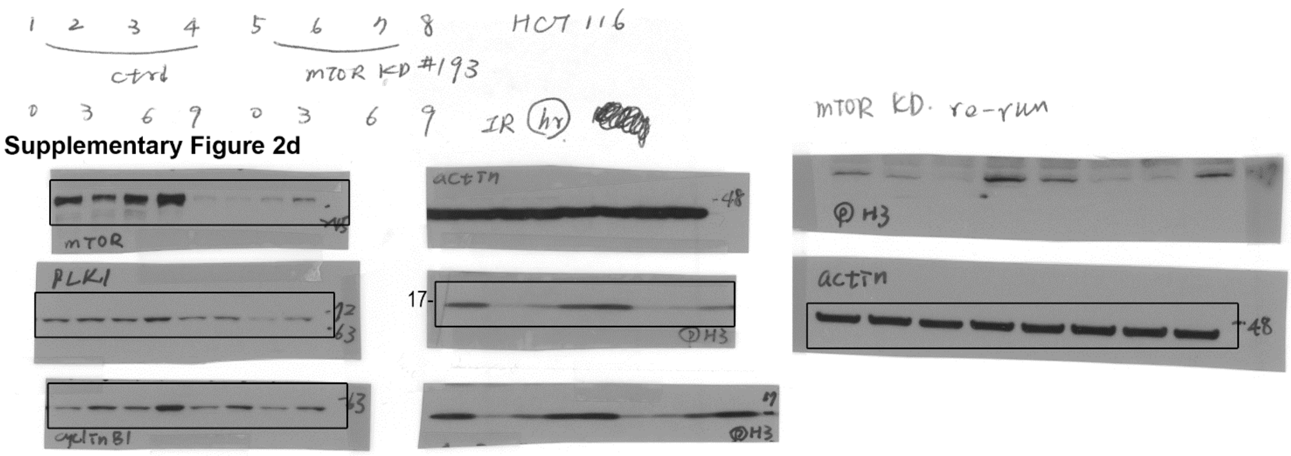

Supplementary Figure 3b

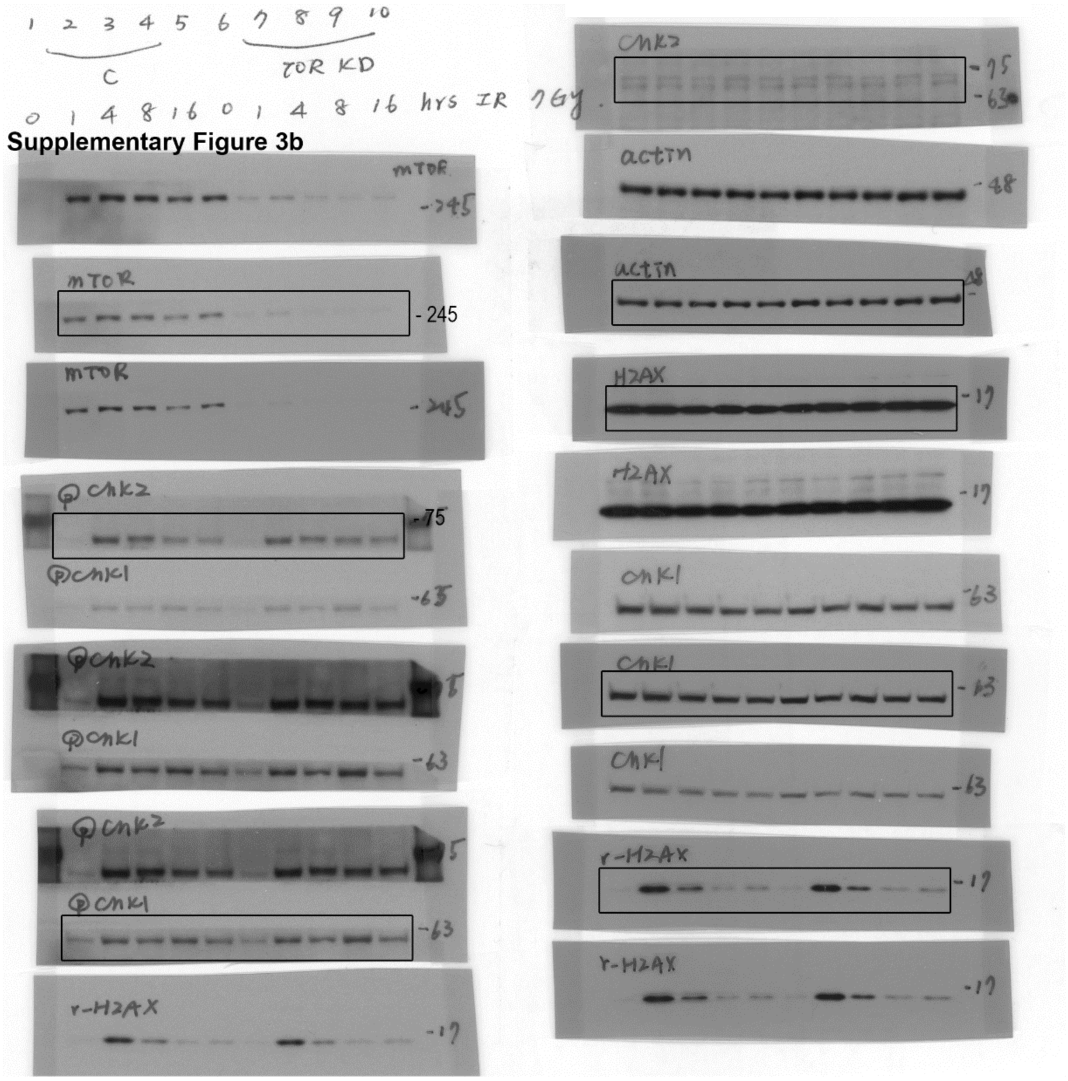

Supplementary Figure 11

Supplementary Figure 3d

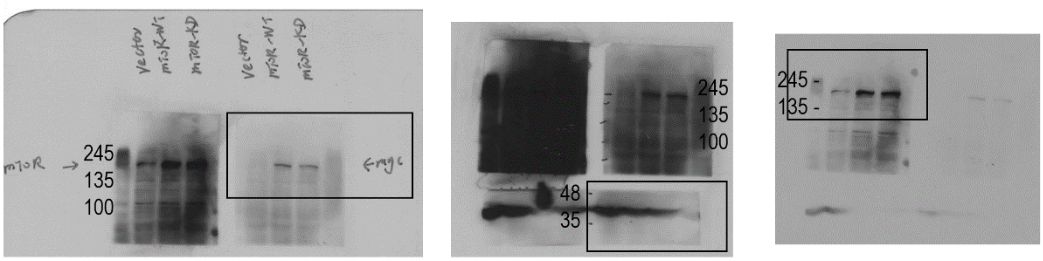

12/30. HCT 116.

1 2 3 4 5 6

0 3 12 0 3 12 hrs

C KDM4B KD IR 7G + Taxol

1 2 3 4

C MK2206 0.1mM 28hrs.

− + − + IR 24 hrs + Taxol

Supplementary Figure 3e

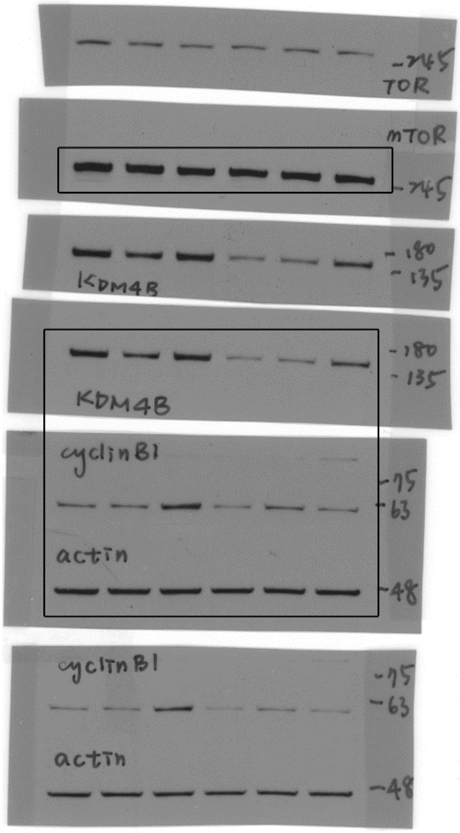

Supplementary Figure 3h

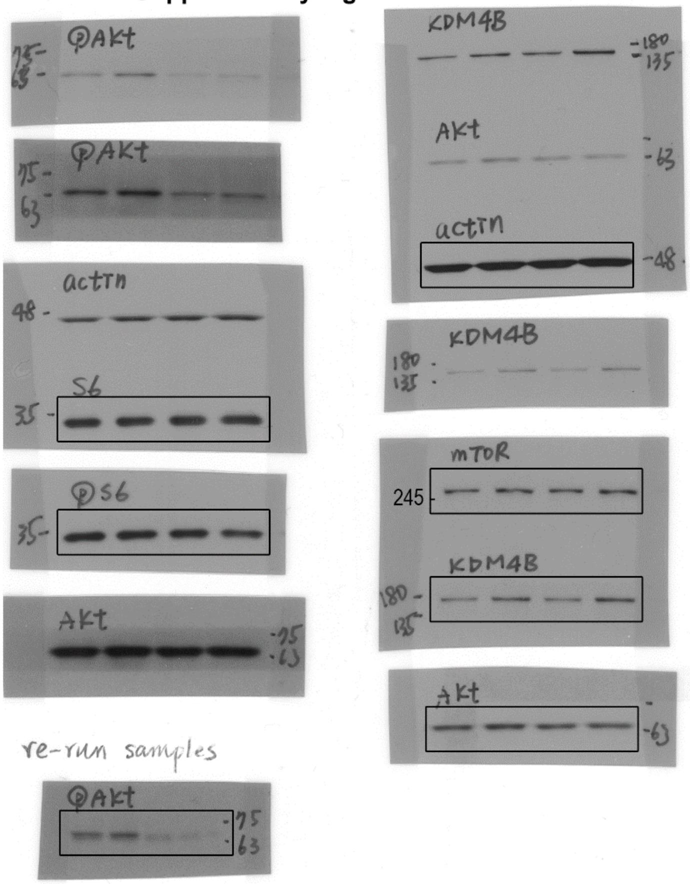

Supplementary Figure 12

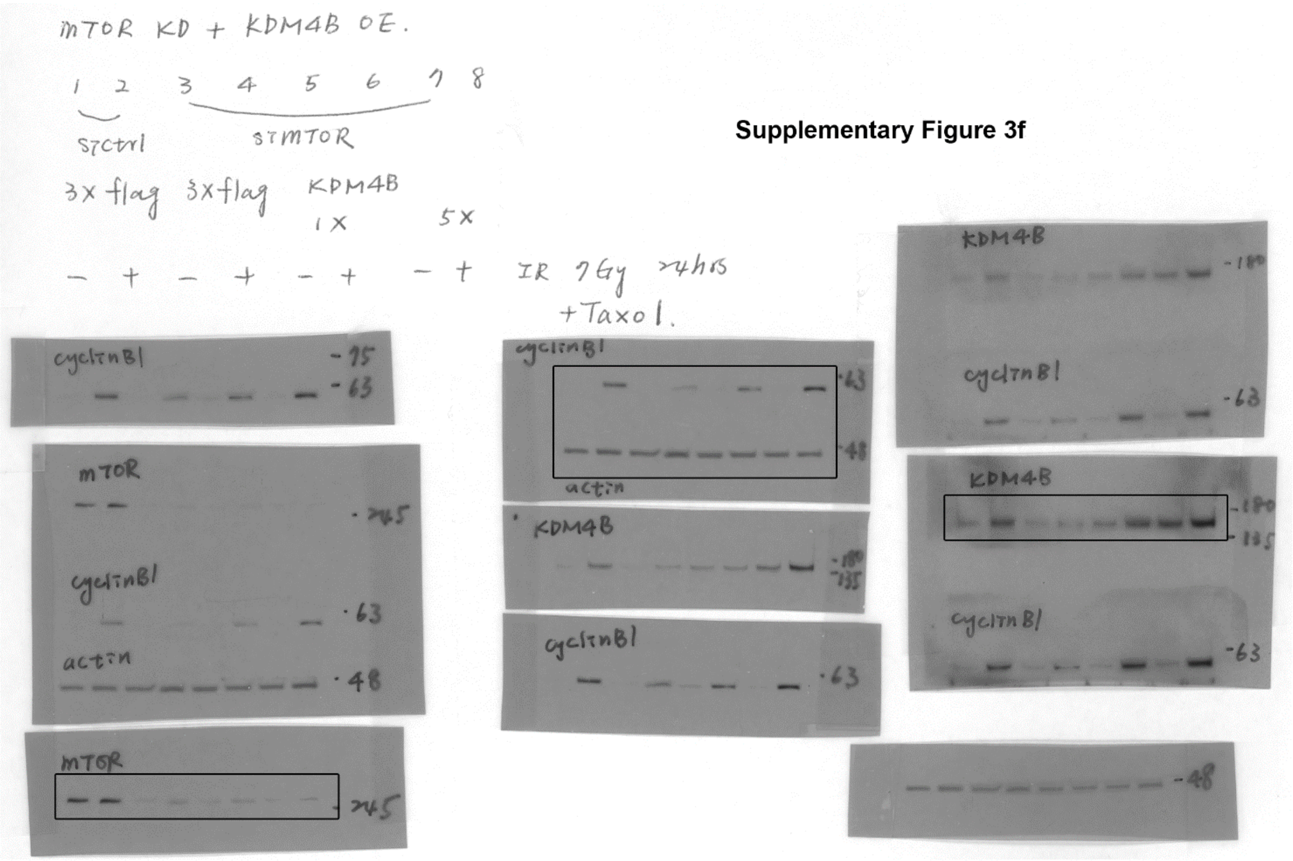

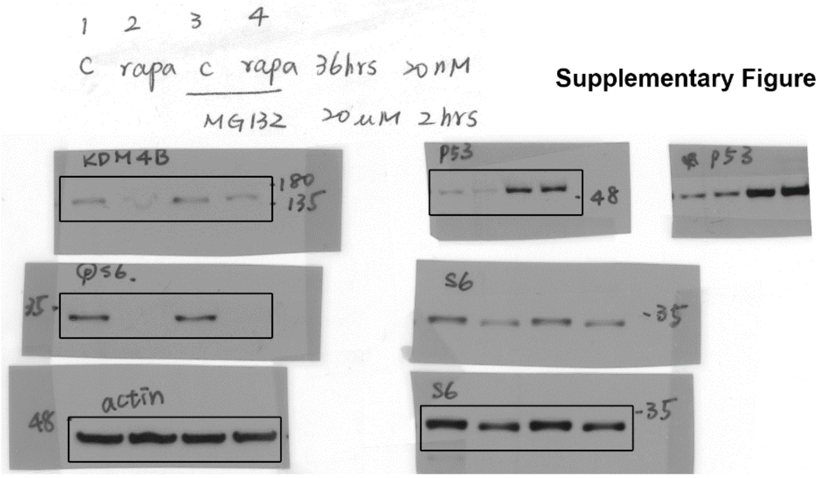

Supplementary Figure 13

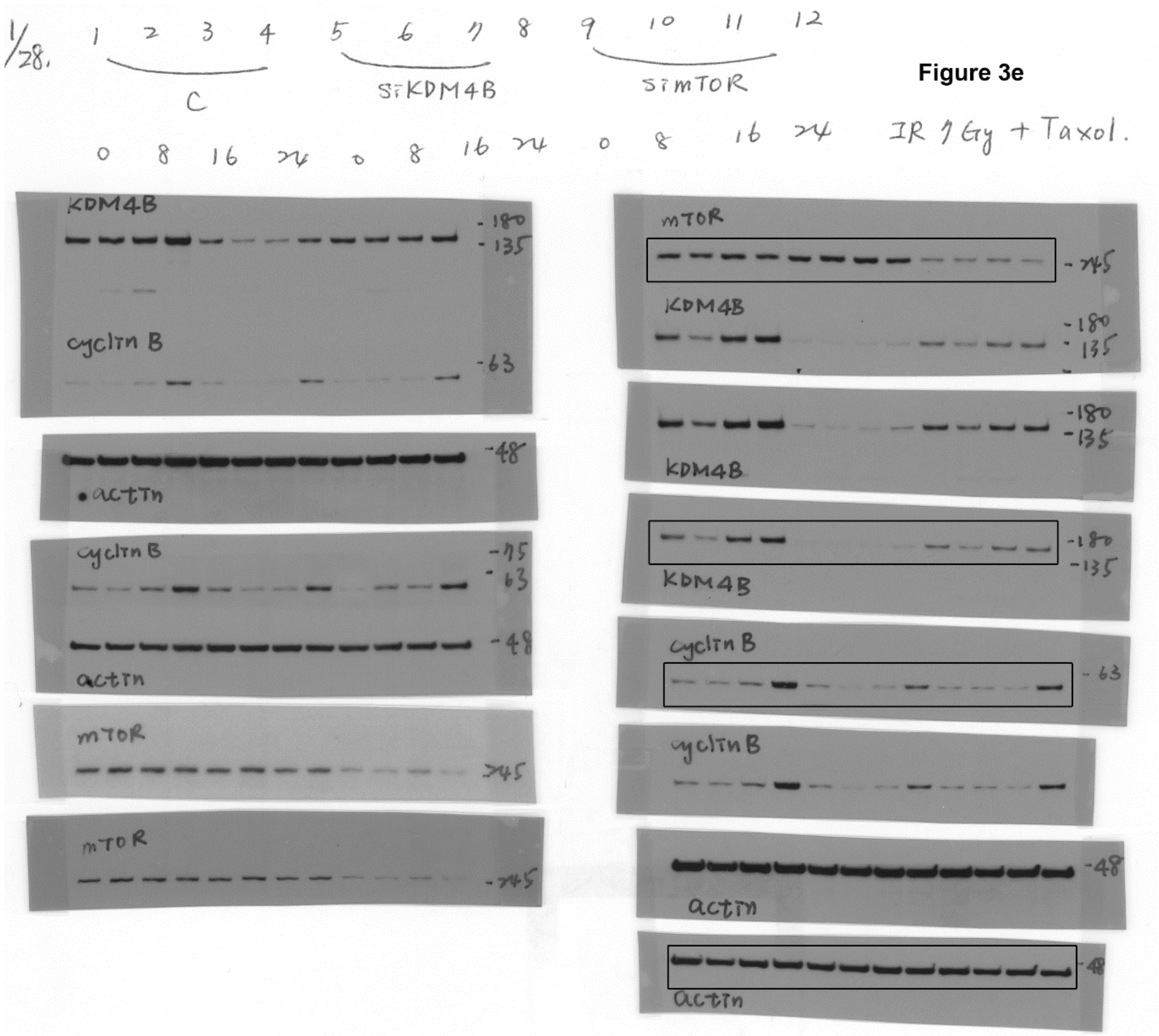

Supplementary Figure 14

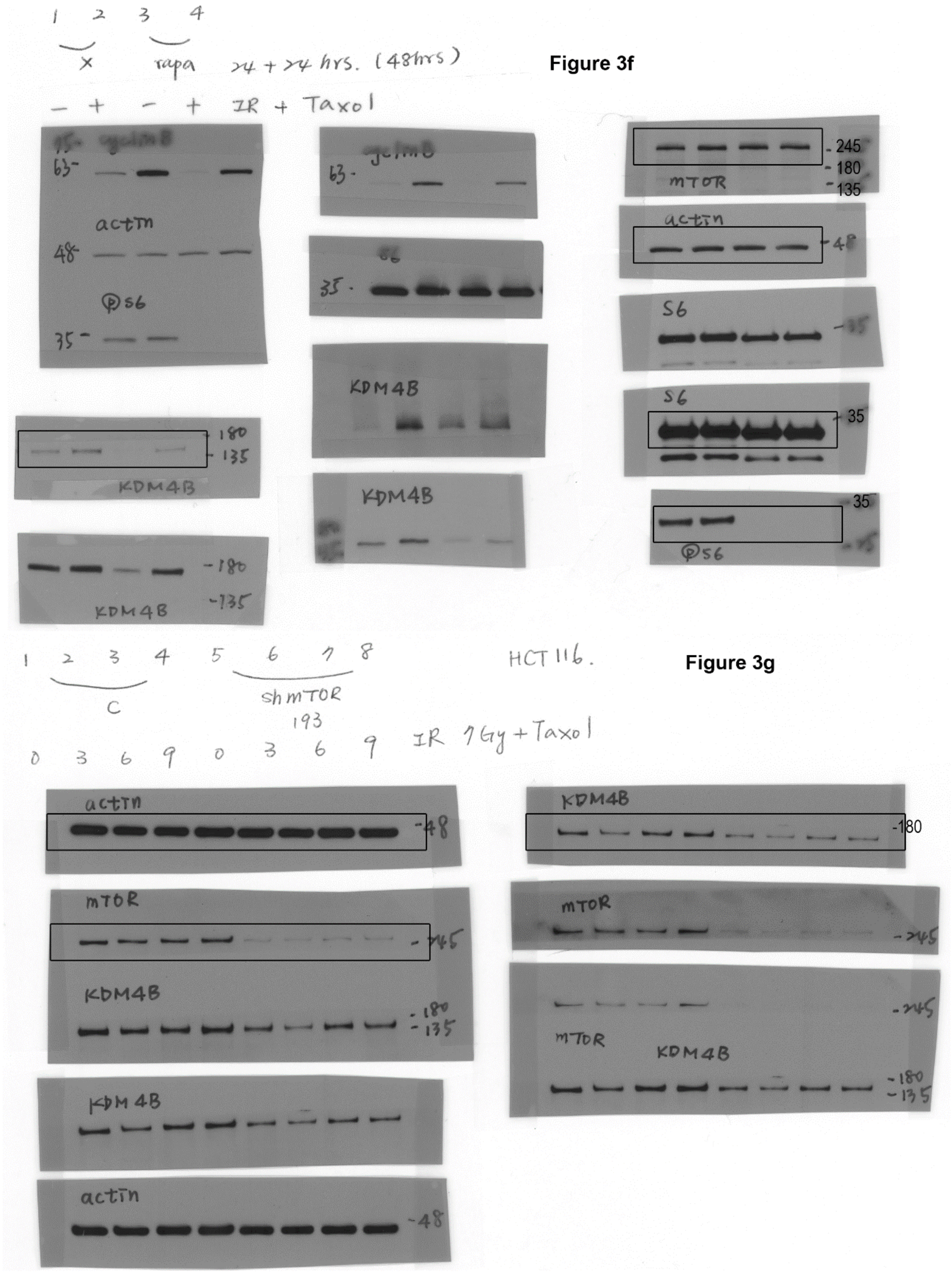

Supplementary Figure 15

Figure 4b

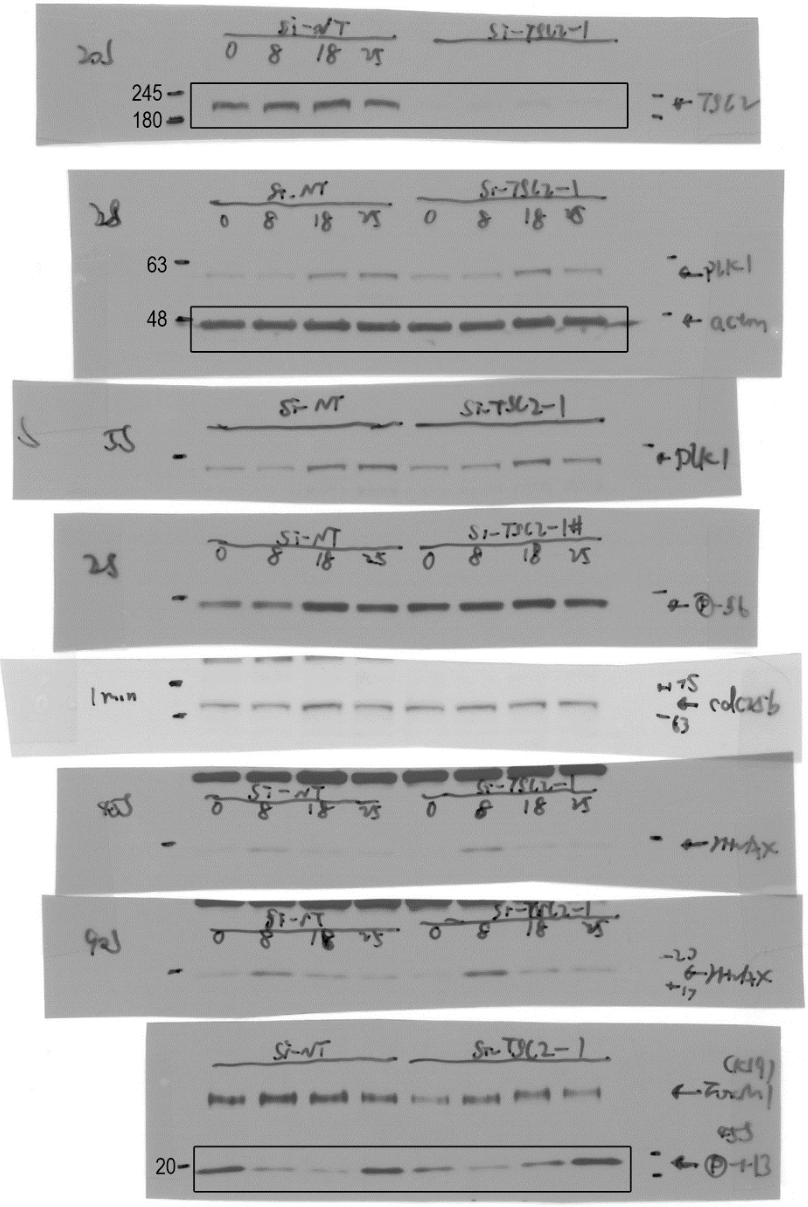

Supplementary Figure 16

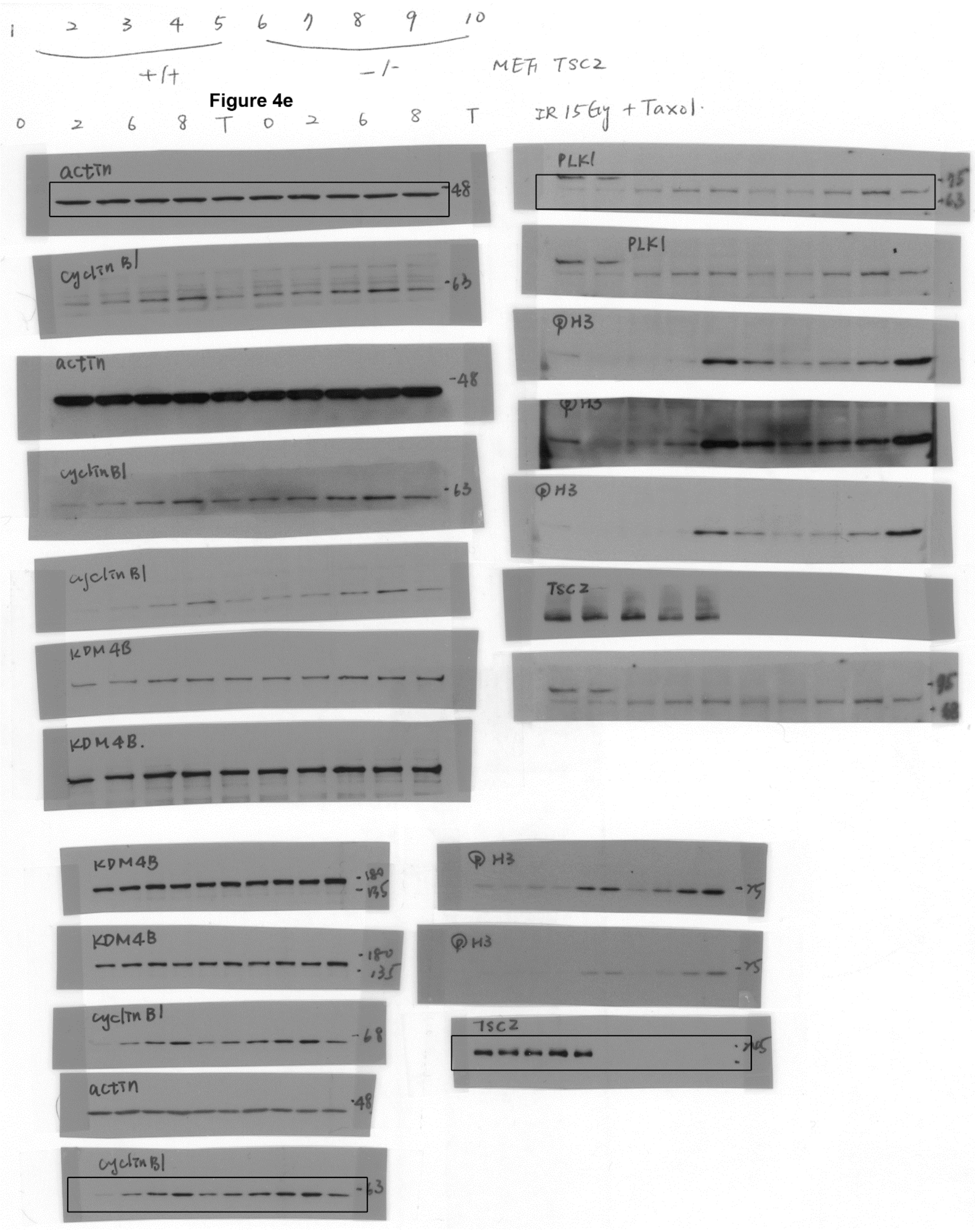

Supplementary Figure 17

Figure 4d

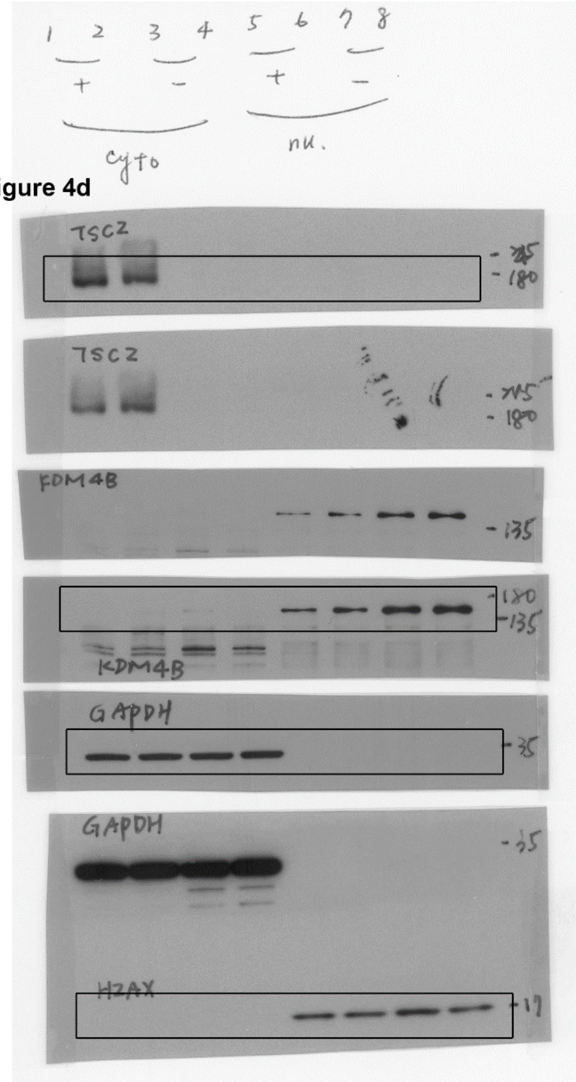

Supplementary Figure 4c

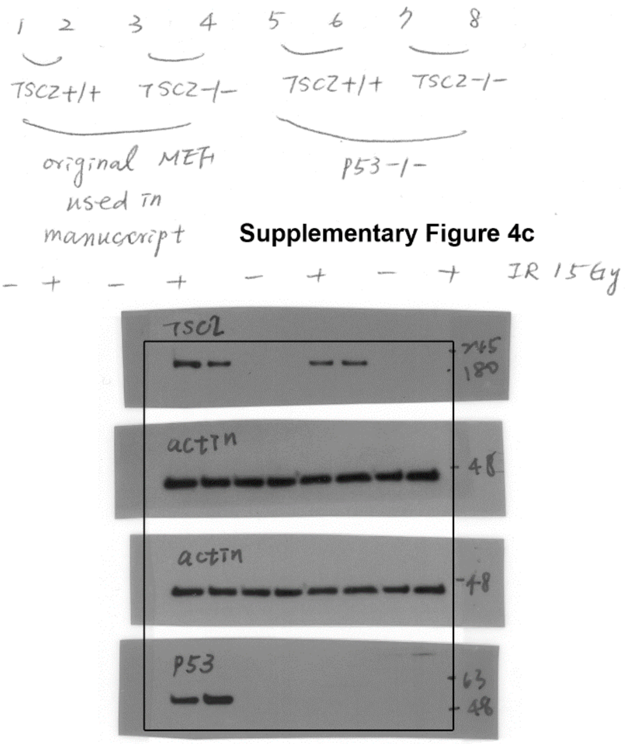

Supplementary Figure 4g

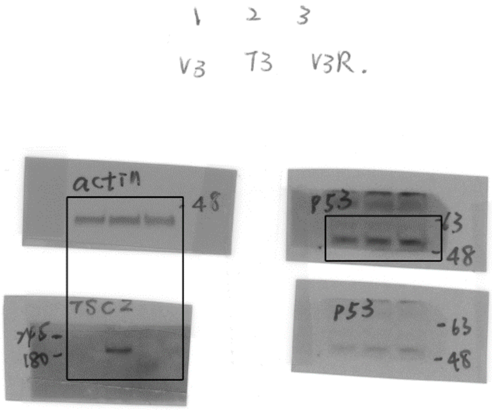

Supplementary Figure 18

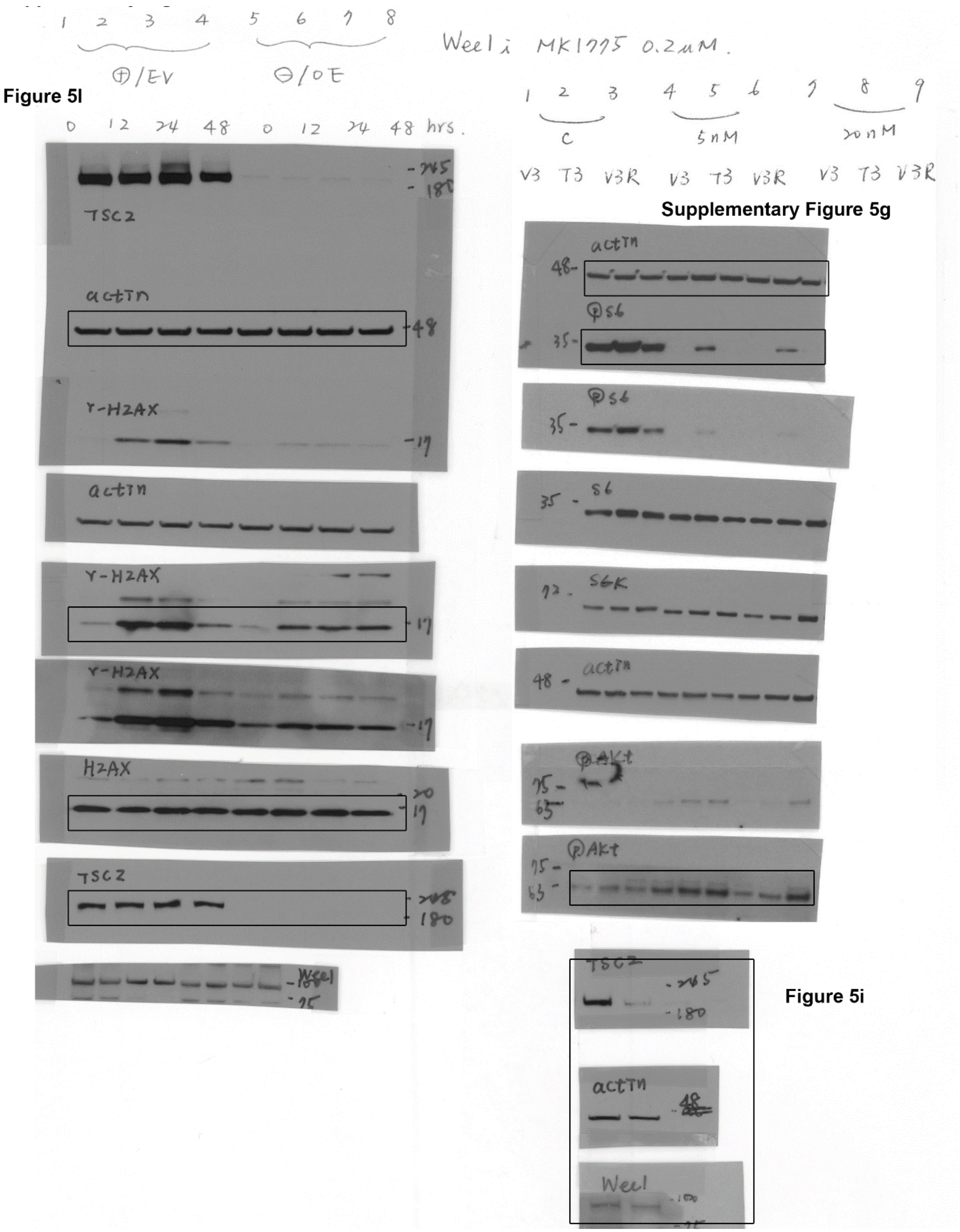

Supplement: Supplementary file 1 — Supplementary Information [file 41467_2018_5639_MOESM1_ESM.pdf]
