# Supplementary material for: Systems biology approach reveals a link between mTORC1 and G2/M DNA damage checkpoint recovery
Source: Nat Commun. 2018 Sep 28;9:3982. doi: 10.1038/s41467-018-05639-x (PMC6162282; doi:10.1038/s41467-018-05639-x)
Supplement: Supplementary file 3 — Description of Additional Supplementary Files [file 41467_2018_5639_MOESM3_ESM.pdf]

Description of Additional Supplementary Files

File Name: Supplementary Data 1
Description: RPPA data from U2OS and HCT116 cells treated with IR

File Name: Supplementary Data 2
Description: Regression and correlation analysis on RPPA data

File Name: Supplementary Data 3
Description: The matrix generated from the IPA network

File Name: Supplementary Data 4
Description: MATLAB results-CCNB1 as the downstream target

File Name: Supplementary Data 5
Description: MATLAB results-CCND1 as the downstream target

File Name: Supplementary Data 6
Description: RPPA data from ELT3 cells treated with rapamycin

File Name: Supplementary Software
Description: R code and MATLAB code used for RPPA data analysis
